# Canadian Notes

**Published:** 1909-11-13

**Authors:** 


### CANADIAN NOTES.

THERE has been recently in Toronto something approaching a storm in a teacup with regard to the manner in which the water supply of the city should be filtered. At the suggestion of some of the municipal authorities the proposal was made that a scheme of water filtration by electricity should be adopted. The method for the purpose of water filtration was initiated by a citizen of the United States, and its principle consists of ozonising the water by means of electrical currents. It is said that the system has been tested with success in the United States and Germany, and a small town in Ontario has just had the method installed. One of the chief points in its favour is the low cost at which it can be worked. A prominent journal in Toronto is strongly in favour of the electrical mode of water filtration being tried in Toronto, in which place water filtration is soon to be commenced on a large scale. But the health authorities of Toronto very wisely say that it is to all intents and purposes an untried method, while slow sand filtration, the method which has been practically decided upon, has proved its merits in all parts of the world. Some mode of effective water filtration is badly needed in Toronto. The sewage is dumped into the bay in the lake, but a short distance from the city, and as the water supply is obtained from the lake in the same direction, if not dangerous to health, it is, at least, unpleasant. Radical reforms, however, are to be introduced shortly, both in the control of the water supply and of the sewage of Toronto.

